# Suberoylanilide hydroxamic acid represses glioma stem-like cells

**DOI:** 10.1186/s12929-016-0296-6

**Published:** 2016-11-18

**Authors:** Che-Chia Hsu, Wen-Chang Chang, Tsung-I Hsu, Jr-Jiun Liu, Shiu-Hwa Yeh, Jia-Yi Wang, Jing-Ping Liou, Chiung-Yuan Ko, Kwang-Yu Chang, Jian-Ying Chuang

**Affiliations:** 1 Graduate Institute of Medical Sciences, Taipei Medical University, Taipei, 11031 Taiwan; 2 Graduate Institute of Neural Regenerative Medicine, Taipei Medical University, 250 Wuxing Street, Taipei, 11031 Taiwan; 3 National Institute of Cancer Research, National Health Research Institutes, 367 Sheng-Li Road, Tainan, 70456 Taiwan; 4 Institute of Biotechnology and Pharmaceutical Research, National Health Research Institutes, Miaoli, 35053 Taiwan; 5 School of Pharmacy, Taipei Medical University, Taipei, 11031 Taiwan; 6 Center for Neurotrauma and Neuroregeneration, Taipei Medical University, Taipei, 11031 Taiwan

**Keywords:** Suberoylanilide hydroxamic acid, GBM stem-like cells, Senescence, Apoptosis, p38, p53

## Abstract

**Background:**

Glioma stem-like cells (GSCs) are proposed to be responsible for high resistance in glioblastoma multiforme (GBM) treatment. In order to find new strategies aimed at reducing GSC stemness and improving GBM patient survival, we investigated the effects and mechanism of a histone deacetylases (HDACs) inhibitor, suberoylanilide hydroxamic acid (SAHA), since HDAC activity has been linked to cancer stem-like cell (CSC) abundance and properties.

**Methods:**

Human GBM cell lines were plated in serum-free suspension cultures allowed for sphere forming and CSC enrichment. Subsequently, upon SAHA treatment, the stemness markers, cell proliferation, and viability of GSCs as well as cellular apoptosis and senescence were examined in order to clarify whether inhibition of GSCs occurs.

**Results:**

We demonstrated that SAHA attenuated cell proliferation and diminished the expression stemness-related markers (CD133 and Bmi1) in GSCs. Furthermore, at high concentrations (more than 5 μM), SAHA triggered apoptosis of GSCs accompanied by increases in both activation of caspase 8- and caspase 9-mediated pathways. Interestingly, we found that a lower dose of SAHA (1 μM and 2.5 μM) inhibited GSCs via cell cycle arrest and induced premature senescence through p53 up-regulation and p38 activation.

**Conclusion:**

SAHA induces apoptosis and functions as a potent modulator of senescence via the p38-p53 pathway in GSCs. Our results provide a perspective on targeting GSCs via SAHA treatment, and suggest that SAHA could be used as a potent agent to overcome drug resistance in GBM patients.

**Electronic supplementary material:**

The online version of this article (doi:10.1186/s12929-016-0296-6) contains supplementary material, which is available to authorized users.

## Background

Glioblastoma multiforme (GBM) has a high recurrence and mortality. The median survival time after a combination of radiotherapy and adjunct temozolomide (TMZ) treatment is only about 14.6 months due to frequent tumor relapse after surgical removal [1]. GBM tumor cells are highly invasive, proliferate rapidly, and easily display resistance to chemotherapy and irradiation. The aggressiveness and high malignancy of GBM are linked to an abundant source of glioma stem-like cells (GSCs) [2]. Cancer stem-like cells (CSCs) are a small population of cancer cells that possess the ability to self-renew and are capable of inducing tumorigenesis [3]. The existence of CSCs has been identified not only in the brain but also in the breast, lung, and colon cancers [4]. GSCs are considered responsible for resistance of GBM to chemotherapy and radiotherapy via their ability to activate the cellular DNA damage response [5] and to resist radiation-induced apoptosis through an increase in free radical scavenging [6]. In order to develop novel, efficient therapeutics against GBM, it is therefore important not only to target GBM cancer cells but also to enhance cytotoxicity of GSCs.

Histone deacetylases (HDACs) are overexpressed in a wide variety of cancers [7], and recent studies indicated that HDACs activities are required for maintaining CSC in breast cancer and head and neck tumors [8, 9]. In order to block their oncogenic potential in malignant disease and their function in CSC, HDAC inhibitors are considered to be applied in both experimental and clinical settings. Suberoylanilide hydroxamic acid (SAHA), a HDAC inhibitor, has been shown to possess cytotoxicity in various cancer cell-types such as T-cell lymphoma [10], breast cancer [11], prostate cancer [12], and glioblastoma cells [10]. A previous study indicated that SAHA elicits anti-cancer effects through the induction of differentiation, apoptosis and cell cycle arrest [7]. However, the pharmacological effects of SAHA on CSCs such as GSCs remain poorly understood.

Cellular senescence is a state of irreversible cell cycle arrest evoked by several oncogenic stimuli. It includes a tumor-suppressive mechanism [13]. Senescence in tumor cells is usually associated with a DNA-damage response and with tumor suppressor pathways such as p53-p21 and p16-pRb [14]. p38 also plays an important regulatory role in inducing cellular senescence, which can be triggered by Ras-Raf activation, oxidative stress, culture stress, or telomere shortening [15]. Furthermore, p38 is shown to be important in mediating oncogene-induced senescence in fibroblasts, indicating a role in tumor suppression [16]. Replicative immortality, which is one of the hallmarks of cancer, is also a potential therapeutic target [17, 18]. Therefore, eliciting cellular senescence or apoptosis could effectively repress immortality of GBM cancer cells and may be important for overcoming poor prognosis in GBM patients [19–21].

In this study, human GBM cells were plated in serum-free suspension cultures allowed for GSC enrichment [3, 22, 23]. We examined the effects of SAHA on cell proliferation, viability, and stem cell-related markers expression in GSCs. In addition, we studied GSCs reduction induced by SAHA through the mechanism of cellular apoptosis and senescence by performing flow cytometry analysis and senescence-associated β-galactosidase (SA-β-gal) assay respectively. By examining the signaling pathway, we further understood the mechanism of action of SAHA. Our results show that SAHA is a potential candidate for treating aggressive and therapy-resistant forms of GBM.

## Methods

### Cell culture

Human glioblastoma U87MG and U373MG cells were obtained from American type culture collection (ATCC). Cells were cultured in Dulbecco’s Modified Eagle’s medium (DMEM) (GIBCO, 12800-017) supplemented with 10% FBS at 37 °C in a humidified atmosphere containing 5% CO_2_. For sphere culture, cells were trypsinized using 0.05% trypsin/EDTA and suspended at a concentration of 1.6 × 10^5^ cells in 6-cm dishes pre-coated with poly-(2-hydroxyethyl methacrylate) (poly-HEMA) for 2–14 days. The cells were cultured in serum-free DMEM/F12 (GIBCO, 12500-062) containing 10 ng/ml EGF (Invitrogen, PHG0311), 10 ng/ml FGF (Invitrogen, PHG0263) and N-2 supplement (GIBICO, 17502-048). After spheroid formation, the spheres were pre-incubated with or without 3 μM SB203580 for 1 h and treated with different doses of SAHA for various time intervals as described in figure legends. The morphology of spheroid bodies was observed using light microscopy.

### Chemicals and antibodies

SAHA was synthesized by Prof. J. P Liou in Taipei Medical University [24] and was dissolved in dimethylsulfoxide (DMSO) (Fisher Scientific, 67-68-5). SB203580, a specific p38 inhibitor, was purchased from Selleckchem (S1076) and dissolved in DMSO. The following commercial antibodies were used in Western blotting and Immunofluorescence: CD133 (Proteintech, 18470-1-AP), Bmi1 (GeneTex, GTX114008), Nanog (GeneTex, GTX100863), Oct3/4 (Santa Cruz, SC5279), Vimentin (GeneTex, GTX100619), Sox2 (GeneTex, GTX101507), tubulin (Proteintech, 66031-1-lg), PARP (Cell Signaling, 9542S), caspase-8 (GeneTex, GTX110723), caspase-9 (Cell Signaling, 9502S), caspase-3 (Cell Signaling, 9661S), cyclin B1 (Santa Cruz, SC245), CDK1 (Santa Cruz, SC8395), p21 (Cell Signaling, 2947S), PCNA (Cell Signaling, 2586S), Phospho-p38 (p-p38, T180/Y182) (Cell Signaling, 9211S), p38 (Cell Singling, 8690S), p53 (Cell Signaling, 2524S), Phospho-p53 (Ser15) (Cell Signaling, 9284S), Phospho-p53 (Ser33) (Cell Signaling, 2526S), and Alexa Fluor 594-conjugated goat anti-rabbit IgG polyclonal antibodies (Invitrogen, A-11037).

### MTT viability/proliferation assay

Cells or dissociated spheroids by trypsin/EDTA were incubated with fresh medium containing MTT (3-(4,5-Dimethylthiazol-2-yl)-2,5-diphenyltetrazolium bromide) reagent (final concentration of 0.5 mg/ml, Bionovas, AM0815-0001) for 1 h at 37 °C. After incubation, the MTT solution was removed and the resultant formazan crystals were dissolved in 200 μl DMSO. The absorbance readings of DMSO extracts were measured at 570 nm using the iMark Microplate Absorbance Reader (Bio-Rad).

### Western blotting analysis

GSCs were lysed using modified RIPA buffer (50 mM Tris-HCl pH 7.8, 150 mM NaCl, 15 mM EDTA, 0.5% Triton X-100, 0.5% Nonidet P-40 and 0.1% sodium deoxycholate) containing a protease inhibitor cocktail (Roche, 04693116001). The lysates were centrifuged at 13,200 rpm for 20 min at 4 °C and supernatants were collected for quantification of proteins by Micro BCA Protein Assay Kit (Thermo scientific, 23235). Equal amounts of proteins from each sample (10–20 μg) were separated by 10–12% SDS-PAGE and transferred onto a PVDF membrane (Bio-RAD, 162-0177). The membrane was blocked with TBST buffer containing 5% nonfat milk, and then was incubated overnight with appropriate primary antibodies at 4 °C. Finally, the membranes were incubated with horseradish peroxidase-conjugated secondary antibodies. Proteins were detected using an ECL kit (GE Healthcare, RPN2106) and visualized on a Kodak autoradiography film.

### Immunofluorescence analysis

Cells were fixed with 4% paraformaldehyde for 20 min, blocked with 1% BSA in PBS for 1 h, and incubated with 1:100-diluted primary antibodies for 4 h at 4 °C. Next, the cells were washed with PBS and incubated with 1:200-diluted Alexa Fluor 594-conjugated second antibodies for 1 h at 4 °C. Finally, the cells were mounted in glycerol containing 4’-6-diamidino-2-phenylindole (DAPI), and examined using a fluorescence microscope (Leica STP6000).

### Senescence-associated β-galactosidase (SA-β-gal) assay

Spheroid GSCs were fixed with 1× fixation buffer provided in an SA-β-gal assay kit (Cell signaling, 9860) for 10–15 min. After fixation, the cells were centrifuged at 800 rpm at room temperature for 3 min, following by manufacturer’s guidelines. For each condition, three bright-field micrographs were captured and counted in three randomly chosen fields. All cultures were performed in triplicate and represented by three independent experiments.

### Soft agar colony formation assay

To examine anchorage-independent growth, trypsinized spheres (2 × 10^4^ cells/ml) were suspended in 0.4% SeaKem LE Agarose (Lonza, 50004) with serum-free DMEM/F12 containing 10 ng/ml EGF, 10 ng/ml FGF and N-2 supplement. This suspension formed the upper layer, which was poured onto a lower layer formed of 0.8% agarose in a composition otherwise identical to the upper layer. The layers with the cell suspension were poured into 6-well plates. The cells were incubated at 37 °C in a 5% CO_2_ atmosphere. After 14–30 days, the cells were stained with 0.05% crystal violet, and the colonies were photographed and counted in three randomly chosen fields. All cultures were performed in triplicate and represented by three independent experiments.

### Annexin V-PI staining

The spheres were centrifuged at 800 rpm for 3 min and trypsinized with 0.05% trypsin/EDTA in order to resuspend them. An FITC Annexin V Apoptosis Detection Kit (BD Pharmingen, 556547) was employed according to the manufacturer’s guidelines. Flow cytometry was carried out using a BD FACSCalibur Flow Cytometer.

### Real-time PCR

Total RNA was isolated by TRIzol (Thermo Fisher Scientific, 15596026), and 3 μg of total RNA were subjected to SuperScript II Reverse Transcriptase PCR (Thermo Fisher Scientific, 18064014). The expression of p53 mRNA was determined using 2 × SYBR real time master mix (Applied Biosystems) and primers specific to p53 (F: 5'- CTTTGAGGTGCGTGTTTGTG; R: 5'- GTGGTTTCTTCTTTGGCTGG), or the control gene GAPDH (F: 5’- GAAGGTGAAGGTCGGAGTC; R: 5’- GAAGATGGTGATGGGATTC). SYBR green fluorescence was then monitored using an ABI 7000 Sequence Detection System (Applied Biosystems).

### Statistical analysis

The data collected from each experiment, containing both experimental and control groups, were reported as means while error bars represent the standard deviation (S.D.). One-way analysis of variance and an unpaired Student’s *t*-test were performed to analyze the differences between groups using the GraphPad Prism 5 program. *, *P* < 0.05; **, *P* < 0.01 are considered statistically significant. The quantification of protein expression was done using band analysis tools in imageLab software 5.2.1 (Bio-RAD). The band intensities obtained from Western blotting were quantified by subtracting background density and normalizing against the loading control.

## Results

### SAHA suppresses cell viability/proliferation and stemness-related markers in GSCs

**Fig. 1:**
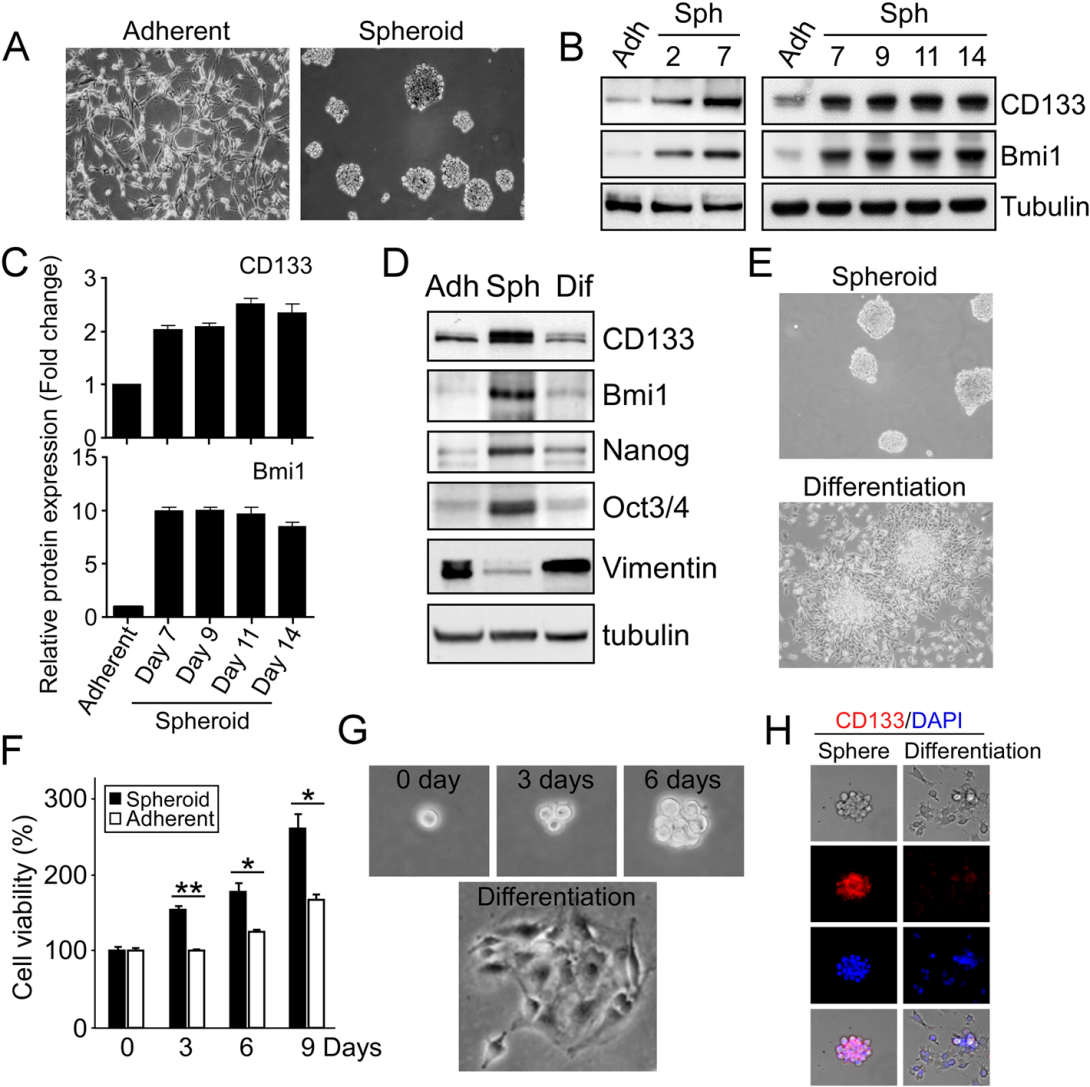
Serum-free suspension culture induces the enrichment of Glioma stem-like cells (GSCs). **a** and **b** GSCs from U87MG GBM cells were grown in serum-free DMEM/F12 medium/suspension culture for 2–14 days. Sphere morphology (2 weeks) was photographed by light microscopy and spheroids (2–14 days) were harvested and subjected to Western blotting using indicated antibodies. **c** The protein expression levels were quantified by imageLab software 5.2.1 (Bio-RAD). The protein expression of U87MG parental (Adherent) was defined as 1 and other values were accordingly normalized. **d** and **e** U87MG spheres were reattached to without poly-HEMA-coated plates in a serum-containing medium for 1 day, in order to induce GSCs differentiation. The levels of stem cell-related markers and Vimentin, an astrocytic marker, in parental cells (Adh), GSCs (Sph), and differentiated GSCs (Dif) were measured via Western blot with antibodies as indicated (**d**). Representative images are shown in (**e**). **f** U87MG adherent cells and dissociated spheroids by trypsin/EDTA were plated at a density of 3 x 10^2^ cells/well. After 0, 3, 6, and 9 days of culture, cell proliferation capacity was determined by using MTT assay. Bar graphs represent means ± standard errors of the means (SEM) from three independent experiments (*t*-test: *p < 0.05; **p < 0.01). **g** A single cell isolated from primary spheroids was cultured in a 96-well dish. The propagation of a secondary spheroid formation was recorded at day 0, 3, and 6, separately (*upper panels*). Subsequently, this secondary spheroid was reattached to plates to induce cellular differentiation (*lower panel*). **h** Immunofluorescence images of CD133 staining in the secondary spheroid and differentiated cells. The nuclei were stained with DAPI (*blue*)

To elucidate the effects of SAHA on GSCs, we used sphere cultures to induce spheroid bodies formation from U87MG (Fig. 1a) and U373MG (Additional file 1: Figure S1A) GBM cells, since a spheroid environment can be employed to enrich CSCs [25, 26]. After 2–14 days of incubation, stem cell-related properties in the spheroids were further examined via stemness-related markers, such as CD133, Bmi1, Nanog, Oct3/4, and Sox2. As shown in Fig. 1b to d and Additional file 1: Figure S1B, the formation of spheroids was in accordance with increased stemness-related protein expression, accompanied by a decrease in astrocyte markers (Vimentin) (Fig. 1d). The spheres were then reattached to plates in serum-containing medium to induce cellular differentiation (Fig. 1e), resulting in decreased levels of stem cell-related markers and an increase in Vimentin (Fig. 1d). Furthermore, we examined tumor proliferation at limiting cell numbers (3 x 10^2^ cell per well) between U87MG adherent cells and spheroids. The result as shown in Fig. 1f demonstrated sphere cells displayed high levels of proliferation potential. Since stem cells are defined as being capable of both self-renewal and differentiation, we further studied generation of the secondary spheres from a single U87MG GSC, as well as induced cell differentiation after the spheroid body formation. As shown in Fig. 1g and h, a single cell from spheroids was able to generate a new sphere and to differentiate into more mature GBM cells. These findings indicate the GBM sphere cells with the stemness-related properties.

**Fig. 2:**
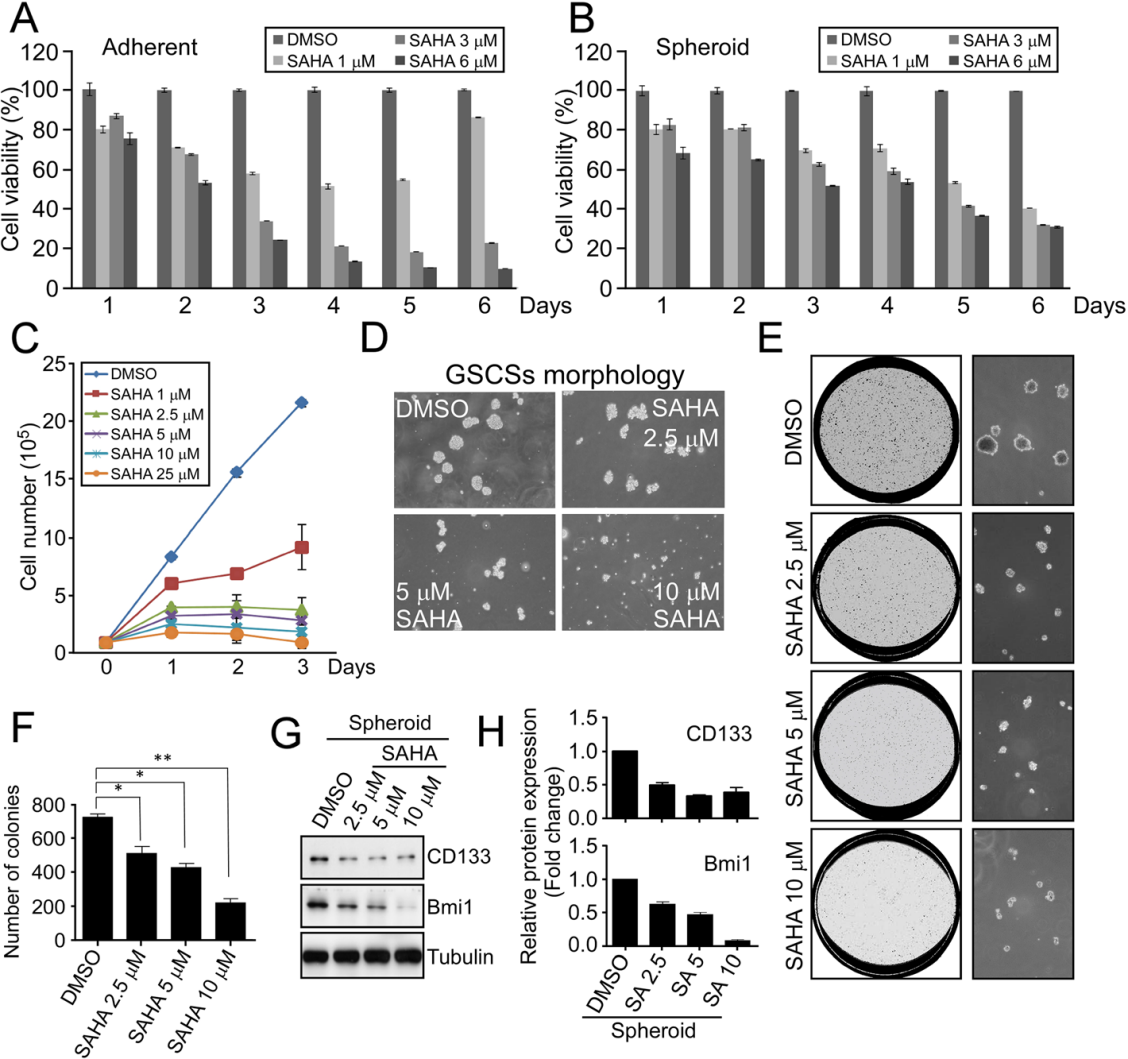
SAHA suppresses cell viability and diminishes the expression stemness-related markers in GSCs. **a** and **b** U87MG adherent cells and spheroids were treated with DMSO or different doses of SAHA (0, 3, 6 μM) for various time intervals (1 to 6 days). After treatment, cell viability was assessed using colorimetric MTT assay. **c** U87MG spheroids were treated with different doses of SAHA as indicated for various time intervals (0, 1, 2, 3 days). After treatment, the cell proliferation was examined by performing cell counting (Trypan blue exclusion) assay. **d** Photomicrographs of GSCSs treated with different dosages of SAHA were randomly selected in microscopic fields. **e** and **f** Cells from dissociated GSCs via trypsinization were grown in soft agar followed by treatment with SAHA at different doses every 48 h. After 14–30 days of incubation at 37 °C, colonies arose from these single cells were photographed randomly and counted in the three fields chosen. The histogram of average colony numbers was obtained by performing the experiment in triplicate; bar, S.D.; *p < 0.05; **p < 0.01. **g** and **h** GSCs after 48 h of SAHA treatment were harvested and the protein expression levels of stemness markers were analyzed by Western blotting with indicated antibodies. The protein levels were quantified by imageLab software 5.2.1 (Bio-RAD)

We subsequently investigated the effects of SAHA on GBM adherent cells and GSCs, and results showed that SAHA treatment appeared to significantly inhibit cell viability in both adherent and GSCs (Fig. 2a, b and Additional file 1: Figure S1C, 1D) as well as to suppress cell growth and sphere formation (Fig. 2c and d). Additionally, since colony formation of single cell by growth in soft agar is indicated reflecting the self-renewing capacity of CSCs [27], we performed a soft agar assay to further examine the effects of SAHA on clonogenicity of GSCs. As expected, SAHA was found to suppress colony formation of cells from dissociated GSCs in a dose-dependent manner (Fig. 2e and f). Furthermore, stemness-related markers such as CD133 and Bmi1 were also decreased by SAHA (Fig. 2g and h). Altogether, the findings suggest that ell viability/proliferation and stemness-related features of GSCs are suppressed by treatment with SAHA.

### SAHA induces apoptosis in U87MG GSCs

**Fig. 3:**
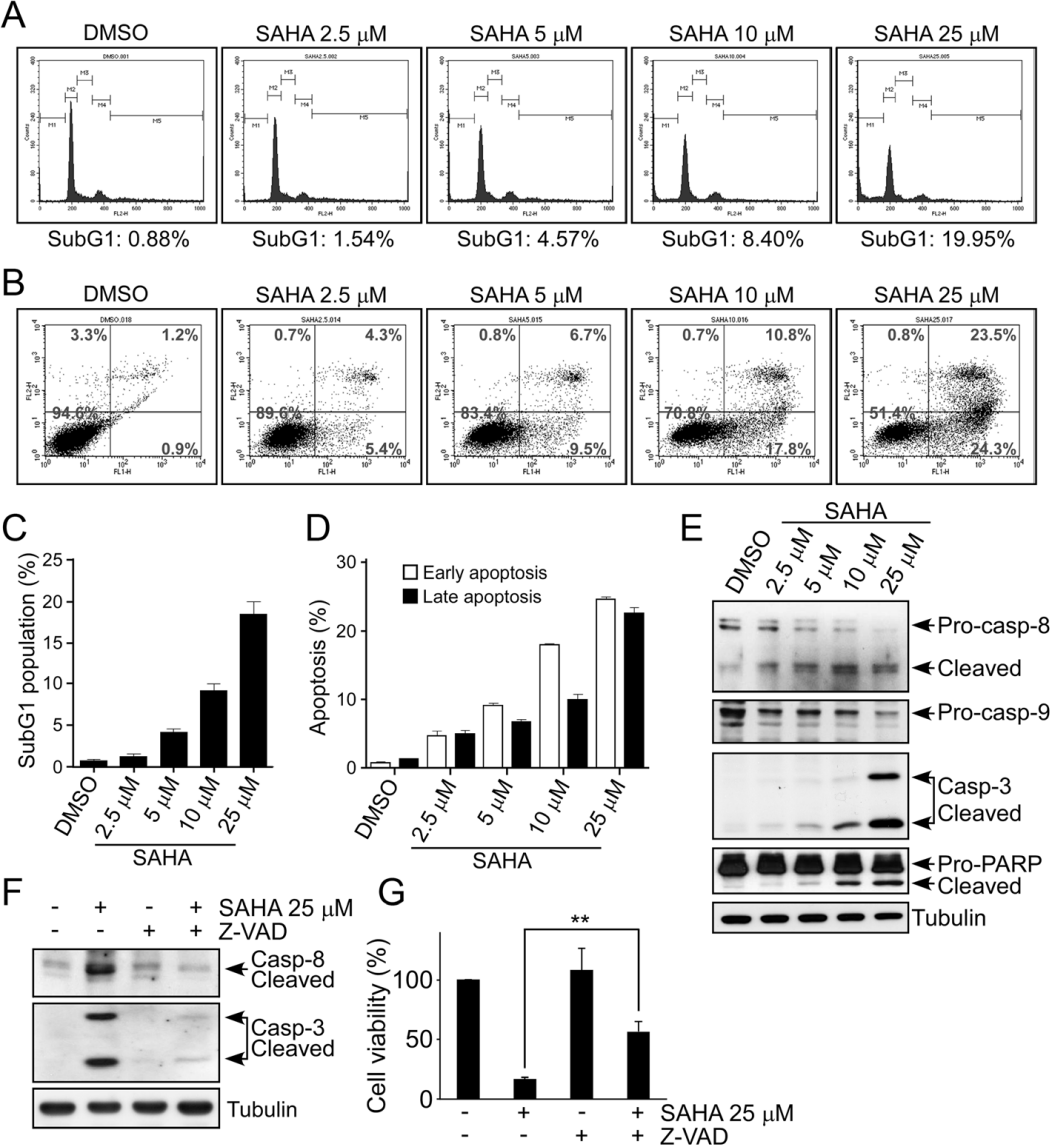
SAHA induces apoptosis in GSCs. **a** and **b** GSCs from U87MG cells were treated with different doses of SAHA as indicated for 2 days. After treatment, cells were stained with PI alone (**a**) and with both FITC Annexin V and PI (**b**), and apoptotic cell death was measured using flow cytometry. **c** and **d** Percentage of sub-G1, early and late apoptotic cells. Data are presented as mean ± S.D. for experiments performed in triplicate; bar, S.D. **e** Expression of cleaved-caspase-8, −caspase-3, and -PARP as well as pro-caspase-9 were determined by Western blotting. **f** and **g** GSCSs were treated with 25 μM SAHA alone, 20 μM Z-VAD-FMK (Caspase Inhibitor) alone, or SAHA + Z-VAD-FMK for 48 h, following which cell lysates were analyzed via Western blot with indicated antibodies (**f**). **g** Cell viability was also assessed using MTT assay. Bar graphs represent means ± SEM from three independent experiments (*t*-test: **p < 0.01)

To clarify the mechanism by which SAHA treatment attenuates growth and viability of GSCs, we examined whether SAHA induces programmed cell death. Flow cytometry with propidium iodide (PI) staining was performed, and the results indicated that the sub-G1 population representing apoptotic cells was increased in response to 2.5 μM, 5 μM, 10 μM and 25 μM of SAHA treatment (Fig. 3a and c). Moreover, SAHA-induced apoptosis in GSCs was further confirmed using the Annexin V/PI assay. Both early (Annexin V positive and PI negative) and late (Annexin V and PI positive) apoptosis populations were increased upon SAHA treatment in a dose-dependent manner (Fig. 3b and d). Furthermore, the cleaved proteins of apoptosis-related markers were measured by Western blotting. The results, shown in Fig. 3e, revealed that SAHA induced the accumulation of activated caspases and cleaved PARP. However, a further experiment conducted using a pan caspase inhibitor Z-VAD-FMK to reduce caspases activation in GSCs resulted in inhibition of SAHA-induced cell death (Fig. 3f and g). In summary, we confirmed that SAHA simultaneously inhibits cell viability and induces apoptosis at both early and late phases in GSCs.

### SAHA at a low concentration leads to cell cycle arrest in GSCs

**Fig. 4:**
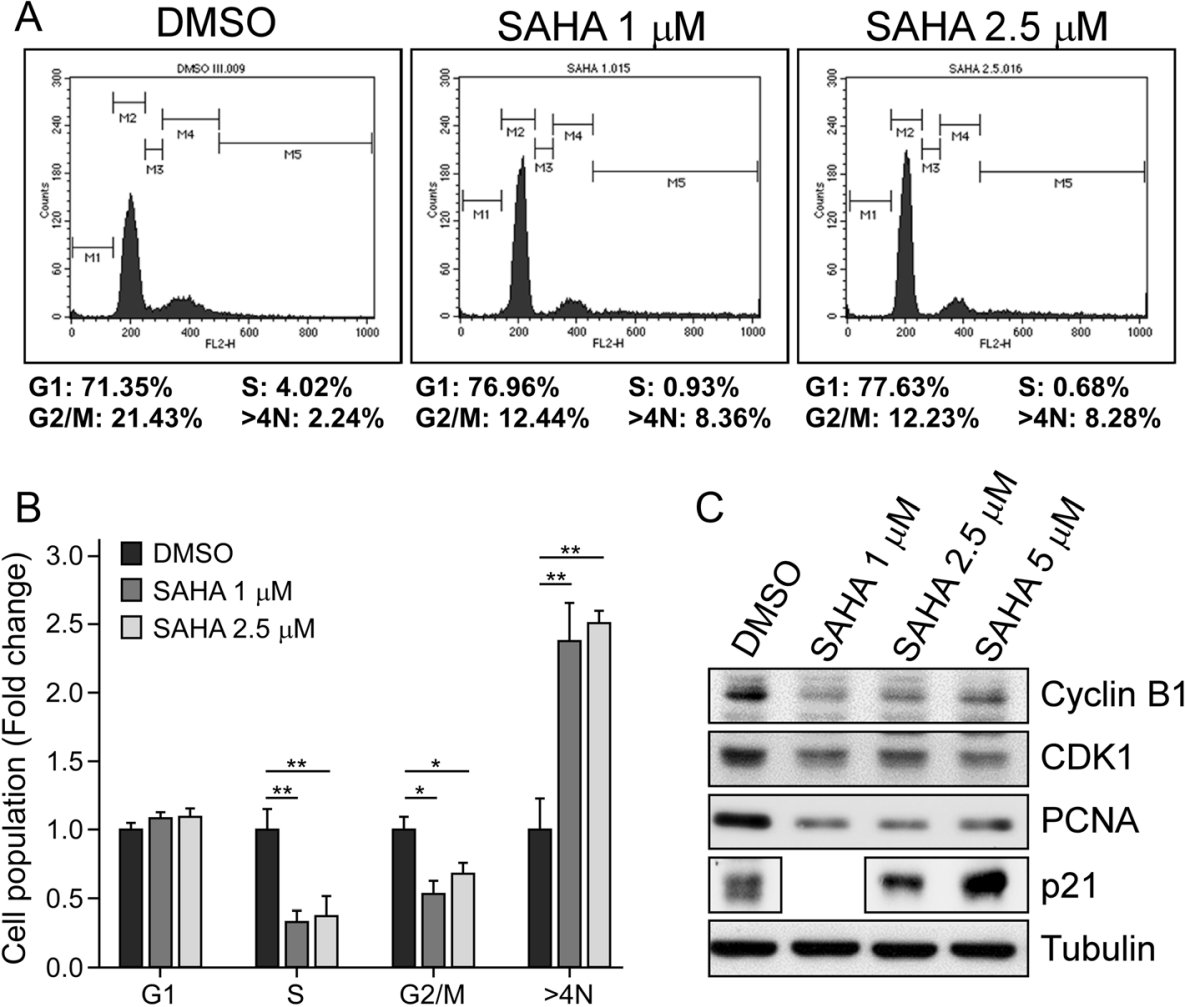
SAHA causes cell cycle arrest of GSCs. **a** GSCs from U87MG cells were treated with SAHA (1 μM and 2.5 μM) for 24 h. The spheres were harvested for PI staining and analyzed by using flow cytometry. **b** Data regarding the population of sub-G1, G1, S, G2/M and > 4 N upon SAHA treatment was presented as the mean ± S.D. of experiments performed in triplicate; bar, S.D.; *p < 0.05, **p < 0.01. **c** The expression levels of cyclin B1, CDK1, PCNA, and p21 were measured by Western blotting. Tubulin was used as a loading control

As shown in Fig. 2, SAHA at a low concentration (2.5 μM) reduced stemness-related markers levels and colony formation, but did not significantly increase apoptotic markers and sub-G1 population in GSCs (Fig. 3). Therefore, we speculate that treatment with a low concentration SAHA causes GSC growth arrest. We assessed the cell cycle of GSCs by flow cytometry, and the results indicated that low doses (1 μM and 2.5 μM) of SAHA caused a decrease in the proportion of S and G2/M cells and a corresponding increase in cells with a DNA content greater than 4 N (Fig. 4a and b). Additionally, the G1 cells were slightly increased in SAHA treatment compared to DMSO-treated controls (Fig. 4a). After finding the significant decrease of the G2/M population, we further examined the expression of G2/M-regulated factors, such as cyclin B1 and CDK1. Their expression levels were significantly downregulated (Fig. 4c). Subsequently, we also found the elevation of p21 and the reduction of PCNA, which may relate to a slight increase in G1 cells and a decrease in S cells respectively after SAHA treatment (Fig. 4c). These results show that SAHA at low concentrations does not trigger apoptosis but interrupts cell cycle progression leading to an increase in polyploid and/or G1 cells in GSCs.

### Low concentrations of SAHA induce premature senescence in GSCs via p38 activation

**Fig. 5:**
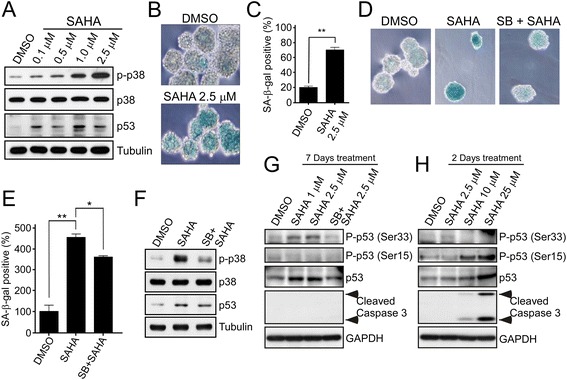
SAHA induces GSCs senescence via activation of the p38-p53 pathway. **a** to **c** GSCs from U87MG cells were collected 7 days after treatment of SAHA at different doses. The cellular lysates were then analyzed by Western blotting with indicated antibodies (**a**). The senescence-associated β-galactosidase (SA-β-gal) activities were then examined by SA-β-gal kit (Cell Signaling, details described in materials and methods). Photomicrographs of spheroid bodies were randomly selected in microscopic fields (**b**) and the percentage of SA-β-gal positive cells was quantified; bar, S.D.; ** *p* < 0.01 (**c**). **d** to **g** GSCs were pre-treated with or without 3 μM SB203580 (SB) for 1 h and then treated with DMSO or with SAHA (1 μM in G, 2.5 μM in **d** to **g**) for 7 days. After treatment, cellular senescence was measured by the detection of SA-β-gal activity (**d**). The percentage of SA-β-gal positive cells was quantified; bar, S.D., *, *P* < 0.05, **, *P* < 0.01 (**e**). The protein lysates were analyzed by Western blotting with indicated antibodies (**f** and **g**). **h** GSCSs were treated with DMSO or with different doses of SAHA as indicated for 2 days. After treatment, the protein lysates were analyzed by Western blotting

Since progressive accumulation of G1 tetraploidy or polyploidy is a unique senescence phenotype [28], we investigated whether premature senescence was induced by SAHA treatment. By examining the signals involved in regulation of cellular senescence, we verified that levels of p53 protein and p38 phosphorylation were up-regulated 7 days after SAHA treatment in a dose-dependent manner (Fig. 5a). Subsequently, we measured the activity of SA-β-gal, which is a direct marker of cellular senescence. We found that GSCs displayed high SA-β-gal activity upon SAHA treatment (Fig. 5b), and the quantification of SA-β-gal positive cells using light microscopy showed about 70% of spheroid bodies becoming senescent (Fig. 5c). The activated p38 signaling pathway has been shown to be responsible for senescence induction in the past [15, 29]. To investigate whether SAHA triggers cellular senescence via p38 activation, we used a p38-specific inhibitor, SB203580, and found that pre-treatment with SB203580 resulted in attenuation of SAHA-induced SA-β-gal activity (Fig. 5d and e). Furthermore, low-dose SAHA-elicited phosphorylation of p38 at Thr180/Tyr182, phosphorylation of p53 at Ser33, and p53 protein levels were also significantly attenuated by SB203580 (Fig. 5f and g). To examine cellular apoptosis by the detection of caspase-3 activity, we found that low doses of SAHA didn’t correlate with apoptosis occurrence (Fig. 5g and h). However, the treatment of short-term and high-dose SAHA induced p53 phosphorylation at Ser15 instead of Ser33 as well as caspase-3 activation (Fig. 5h). These results demonstrate that p38-mediated activation of p53 through phosphorylation at Ser33 is essential for low-dose SAHA-induced senescence in GSCSs, but not affecting high-dose SAHA-induced cell death.

## Discussion

**Fig. 6:**
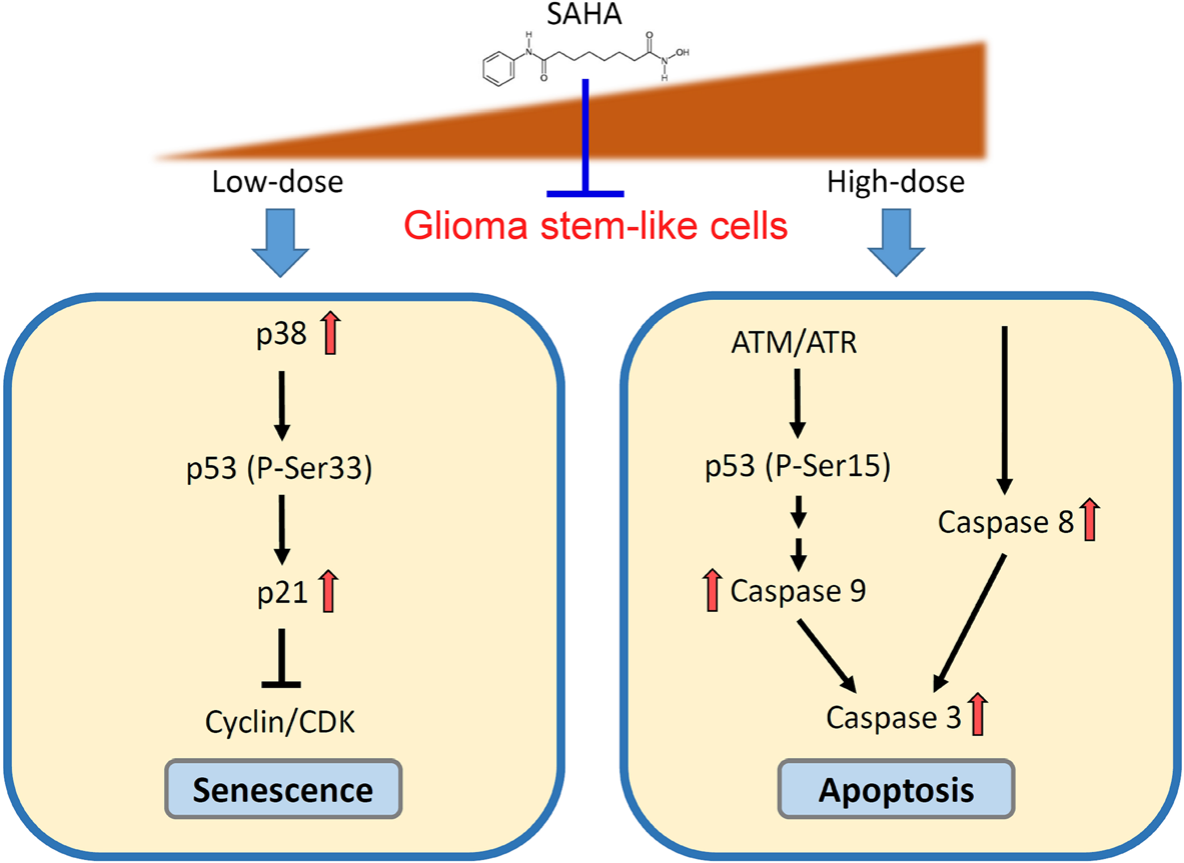
A diagram illustrates the signaling mechanisms of how SAHA may be inhibiting GSCs. Low-dose of SAHA increases both p53 protein and its phosphorylation at Ser33 via p38 activation, leading to a rise in cell cycle checkpoint activation and cellular sensecence, but high-dose of SAHA induces p53 Ser15 phosphorylation and caspase-mediated involving both the caspase-8 and the caspase-9 pathways in GSCs

The clinical treatment of GBM usually begins with surgery, followed by radiation therapy and chemotherapy. However, the median time of survival for GBM patients is only about 1 year and the 5-year survival rate is less than 5% [30]. An abundance of CSCs may contribute to the therapeutic resistance of GBM and a consequently high mortality rate [2, 5, 31, 32]. As shown in Additional file 1: Figure S2, the result of immunohistochemistry staining verified that the stemness proteins CD133 and Bmi1 were indeed increased in TMZ-resistant tumors in xenograft models. In order to reduce therapeutic resistance, development of a novel drug targeting GSCs is important. In our study, we used sphere cultures-induced GSCs and found that SAHA treatment inhibited cell viability/proliferation and stemness-related markers expression in GSCs. Although several studies have demonstrated that caspase-dependent apoptosis is an effective strategy for treating GBM, it has been proven that malignant gliomas develop anti-apoptosis mechanisms to escape tumor cell death [33, 34]. Interestingly, our study revealed that SAHA not only induced apoptotic cell death at higher doses but also induced senescence in GSCs at low concentrations (Fig. 6). Additionally, previous studies indicate that SAHA treatment has some side effects including nausea, vomiting, diarrhea, and anemia [35–37]. To relieve these side effects, a reduced dosage of SAHA treatment is necessary. Altogether, we demonstrated that SAHA inhibits GSCs via different mechanisms at high and low doses. Moreover, and more importantly, a restriction of GSCs at lower doses of SAHA might indicate potential for administration of SAHA, and it may decrease uncomfortable side effects in patients.

Self-renewal in CSCs fuels tumor growth and provides the maintenance of stemness in a small population of cancer cells [3, 38]. If a therapeutic treatment eliminates the non-self-renewing cells, but not the CSCs. In this case, the tumor would shrink, but it would return form the remained CSCs when the therapy is stopped. Therefore, preventing drugs resistance and self-renewal in GSCs are important to avoid GBM recurrence. As shown in Fig. 2, we found that GSCs were inhibited upon SAHA treatment, suggesting that SAHA may overcome therapeutic resistance in GBM by targeting GSCs. Furthermore, the marker Bmi1 is an important factor for the self-renewal and stemness maintenance of CSCs [39]. This suggests that targeting Bmi1 may be an efficient way to prevent cancer recurrence. As shown in Fig. 2, SAHA significantly suppressed Bmi1 expression in a dose-dependent manner, implying that SAHA inhibits GSCs by blocking the self-renewal mechanism. In addition, we have examined the viability between U87MG adherent cells and U87MG spheroids by performing MTT assay, and verified SAHA at higher doses, primarily induces apoptosis, leading to inhibition of cellular proliferation in both cells (Fig. 2a and b). Interestingly, at low-dose treatment of SAHA (1 μM), only spheroids were inhibited well up to 6 days after treatment comparing with U87MG adherent cells. The result implies that GSCs are sensitive to SAHA-induced senescence.

Previous study indicated that p53 plays an essential role in SAHA-induced apoptosis in MCF-7 cells [40], whereas other studies suggested that SAHA shows preferential cytotoxicity in several p53-mutant cancer cells through inhibition of the HDAC6-Hsp90 chaperone axis [41], indicating the complexity of mechanism by which SAHA medicates apoptosis in wild type and p53-deficient tumors. In cancer stem cell-like spheroids, we found that both levels of p53 protein and its phosphorylation at Ser15, but not at Ser33, were increased within 2 days after treatment with high doses (10 μM and 25 μM) of SAHA (Fig. 5h). Especially, these doses of SAHA triggered significant apoptosis in U87MG (p53wt) GBM cells (Fig. 3b). When we further used another malignant GBM cell line, U373MG that expresses mutant p53 protein (R273H mutation, p53R273H), however, a high-dose SAHA (10 μM) inhibited cell viability in U373MG spheroids as well (Additional file 1: Figure S1). These results indicated that SAHA inducing cell death in cancer cells and even in cancer stem cell-like spheroids might be through both p53-dependent and p53-independent apoptosis pathways. A recent study further investigated the mechanism of SAHA-induced apoptosis in couple leukemic cell lines, HL-60 (p53null) and CML-T1 (p53wt) [42]. While leukemic cells were treated with combination of SAHA and decitabine (DAC), a methyltransferase inhibitor, p53-dependent apoptosis is triggered by the upregulation of Puma and p21 in CML-T1 cells, and p53-independent apoptosis is triggered by inhibition of survivin and upregulation of p21 in HL-60 cells. Although this study probably explained the different mechanisms of SAHA-induced cell death between p53null and p53wt cancer cells, in GSCs the roles of p53 after SAHA treatment remains unclear and needs further studies.

Since many post-translational modifications reportedly modulate protein stabilization and transcriptional activation of p53 [43], the rigorous control of these modifications on p53 may determine its functional role under several stresses. Various p53 residues can be phosphorylated by different kinases. For example, phosphorylation at Ser 6, 9, and 15, is carried out by ATM, occurs in response to genotoxic stresses, especially Ser15 of p53 is rapidly phosphorylated after stress in order to represent a priming event for the subsequent series of modifications [44], such as Ser20 phosphorylation by Chk2 and C-terminal acetylation by p300, CBP and P/CAF. Then these secondary modifications prevent the degradation of p53 and increase its apoptotic induction [43, 45]. Furthermore, stressful situations may also elevate p53 phosphorylation at Ser33, by p38 kinase, and at Thr81, by JNKs kinase, all of which are present in Ser/Thr–Pro motifs. These phosphorylated motifs lead to p53 interacting with Pin1 which in turn induces proline isomerization in p53 [46]. Such conformational changes have intense effects on p53 transcriptional activity and protein stability and shows an increase in cell cycle checkpoint activation following genotoxic stress. Therefore, it is evident that only a number of stress-responsive kinases alter p53 at exact sites which recruits Pin1 interaction in order to complete an additional step in the process of activation.

When we treated GBM stem cell-like spheroids with low doses of SAHA (1 μM and 2.5 μM), we found that both p53 protein and its phosphorylation at Ser33 were slight increased on 2 days after SAHA treatment, but they were risen significantly after 1 week of treatment (Fig. 5g and h). However, pharmacological inhibition of p38 kinase attenuated p53 accumulation and its phosphorylation at Ser33, which resulted in a reduction in the senescent phenotype (Fig. 5d to g). In addition, we observed that low doses of SAHA-induced phosphorylated p53 at Ser33 didn’t seem to correlate with apoptosis occurrence by the detection of caspase-3 activity (Fig. 5g and h). Therefore, we summarized the results revealing that low-dose and long-term treatment with SAHA in GBM spheroids induces p53 phosphorylation at Ser33 causing an increase in cell cycle checkpoint activation (Fig. 6), but SAHA in high-dose and short-term treatment induces p53 phosphorylation at Ser15 leading to p53-dependent apoptosis in U87MG spheroids, and even induces p53-independent apoptosis in U373MG spheroids (Fig. 6).

To further understand the regulated mechanism of p53 after SAHA treatment, Real-time PCR was performed and the result showed a slight increase in *p53* mRNA levels after low concentrations treatment of SAHA, but no change at a high-dose (10 μM) treatment (Additional file 1: Figure S3). Combining with the reason that phosphorylation of p53 at multiple sites, including Ser15, Ser20, and Ser33, can protect its protein stability against MDM2-mediated ubiquitination and degradation [43], we think that low-doses SAHA might alter the transcriptional activity and protein stability to trigger p53 up-regulation, but high-doses SAHA rise p53 levels primarily by preventing the protein degradation.

Cellular senescence is a tumor-suppressive mechanism leading to permanent cell cycle arrest. Although SAHA-induced polyploidy, resulting in loss of proliferation and commitment to senescence, has been demonstrated in human colon cancers and breast cancer cells [47], the effects and molecular mechanisms of SAHA in CSCs remained unclear. In our study, we clarified that a reduced dose of SAHA not only increases polyploidy but also decreases S and G2/M cells in GSCs, indicating that SAHA induces GSCs senescence by blocking cell cycle progression as well as via polyploid cell accumulation. Previous study indicated that Cyclin A1 is a p53-induced gene that mediates cycle arrest and polyploidy [48]. Additionally, we used a p38 inhibitor to demonstrate that p38-regulated p53 phosphorylation at Ser33, affecting p53 transcriptional activation and protein accumulation, is essential for SAHA-induced GSC senescence. Crucially, SAHA has been determined capable of crossing the blood-brain barrier at micromolar concentrations [49, 50]. Taken together, the evidence suggests that at lower doses, SAHA may be used as a drug targeting CSCs through the induction of senescence, helping to overcome drug resistance in GBM.

## Conclusions

We demonstrated that SAHA suppressed cell proliferation and diminished stemness properties in GSCs. Administration of high concentrations of SAHA triggered apoptosis of GSCs accompanied by increases in both activation of caspase 8- and caspase 9-mediated pathways. Interestingly, we found that SAHA at a low dose led to cell cycle arrest in GSCs via upregulation of p21, and induced premature senescence through activating p38/p53 pathway. Our results provide a perspective on targeting GSCs via SAHA treatment, and suggest that SAHA could be used as a potent agent to overcome drug resistance in GBM patients.

## Additional file

Additional file 1: Supplementary information includes Figures S1 to S3 and supplementary methods. (DOCX 654 kb)
