# Supplementary material for: Suberoylanilide hydroxamic acid represses glioma stem-like cells
Source: J Biomed Sci. 2016 Nov 18;23:81. doi: 10.1186/s12929-016-0296-6 (PMC5116136; doi:10.1186/s12929-016-0296-6)
Supplement: Additional file 1: — Supplementary information includes Figures S1 to S3 and supplementary methods. (DOCX 654 kb) [file 12929_2016_296_MOESM1_ESM.docx]

**Supplementary figures and legends:**


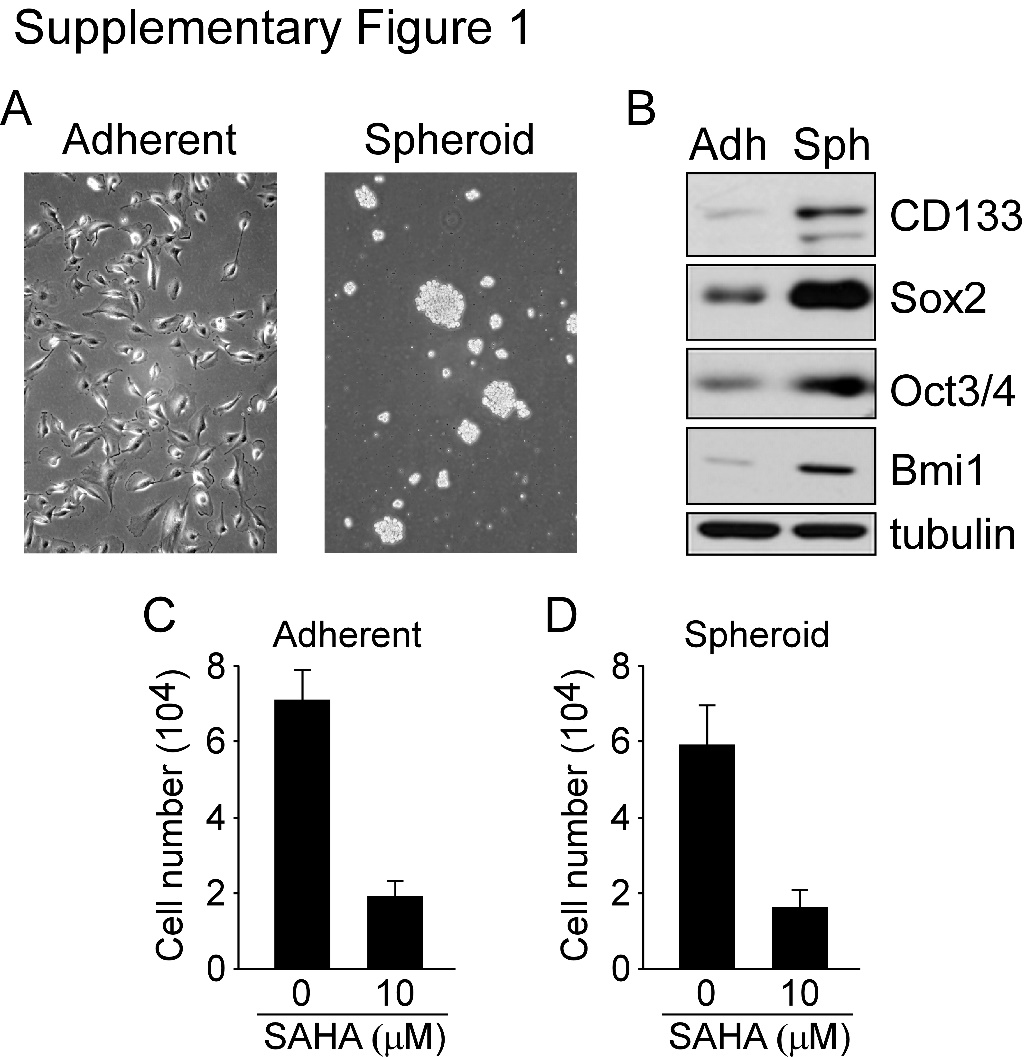


**Supplementary Fig. 1. SAHA suppresses cell viability in U373MG stem cell-like spheroids.** (A and B) The spheroids of glioma stem-like cell (GSC) from U373MG GBM cells were grown in serum-free DMEM/F12 medium/suspension culture for 1–2 weeks. Sphere morphology was photographed by light microscopy and spheroids were harvested and subjected to western blotting using indicated antibodies. (C and D) U373MG adherent cells and spheroids were treated with DMSO or with 10 μM SAHA for 2 days. After treatment, cell viability was assessed using colorimetric MTT assay.


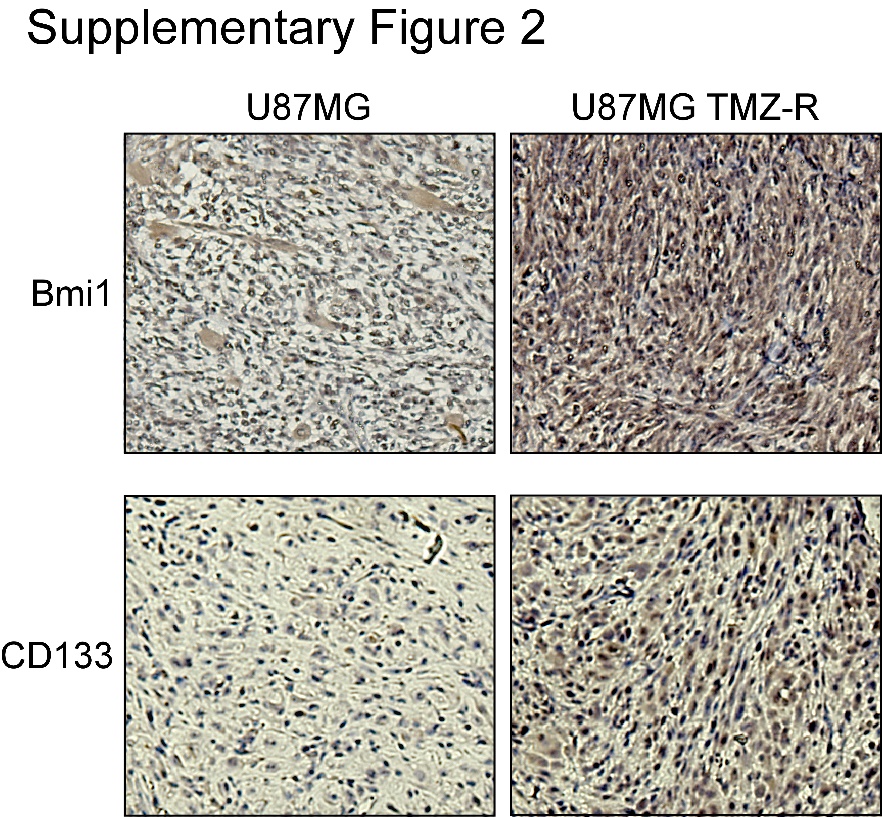


**Supplementary Fig. 2. Stemness-related markers is up-regulated in temozolomide (TMZ)-resistant tumors.** Acquired TMZ-resistant (TMZ-R) tumor model was used for CD133 and Bmi1 IHC staining. TMZ was or was not given continuously after U87MG inoculation until the tumor formed.


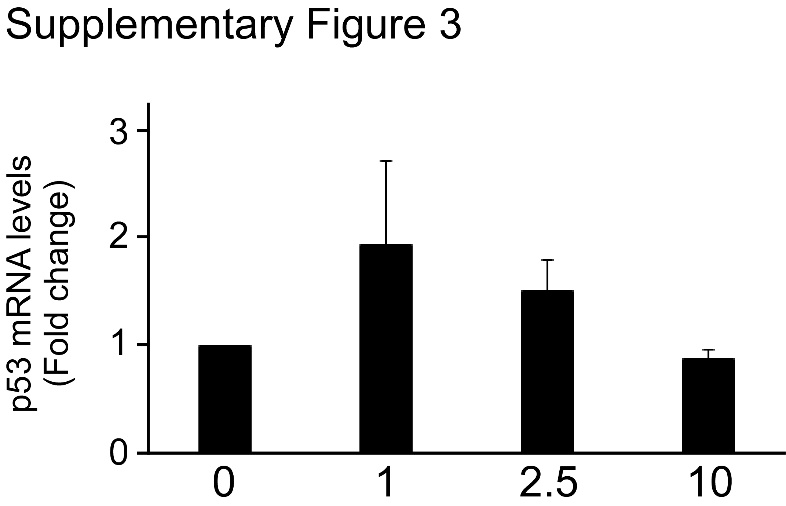


**Supplementary Fig. 3 Low-dose SAHA slightly elevates the levels of p53 mRNA.** GSCs from U87MG cells were treated with different doses of SAHA as indicated for 1 days. After treatment, Total RNA was isolated by TRIzol, and the expression of p53 mRNA was determined using 2 × SYBR real time master mix and primers specific to p53.

**Supplementary** **methods**

**Experimental animals**

Male NOD-SCID mice (6 weeks old, BioLASCO, Taiwan) were maintained at the animal facility of the National Health Research Institutes (NHRI, Taiwan). U87MG cells (2 × 10^6^) were inoculated into the right flank of mice. Tumor volume was measured twice a week based on the following formula from the National Cancer Institute: tumor volume = length × width2 × 3.14/6. When the volume reached 200 mm^3^, animals were randomly divided into two groups: saline control and TMZ treatment. TMZ group was treated with 5 mg/kg TMZ administered orally 5 days a week for 2 weeks. Although the drug significantly reduced tumor volume after treatment, the tumor recovered about 50 days after TMZ treatment.

**Immunohistochemistry (IHC)**

IHC staining were done on 10-μm-thick sections of paraformaldehyde-fixed and paraffin-embedded xenograft tumor tissues. The method of IHC was described previously [1]. The slides were incubated with primary antibody at a dilution of 1:200 after antigen retrieval, and then revealed using the EnVision+ staining kit (DAKO).

**Supplementary** **references**

1. Chuang JY, Wang SA, Yang WB, Yang HC, Hung CY, Su TP, et al. Sp1 phosphorylation by cyclin-dependent kinase 1/cyclin B1 represses its DNA-binding activity during mitosis in cancer cells. Oncogene. 2012;31(47):4946-59.
